# A Novel Approach to Percutaneous Endoscopic Gastrostomy Tube Replacement: Can You Save the Site?

**DOI:** 10.7759/cureus.20718

**Published:** 2021-12-26

**Authors:** Jobin Philipose, Dhineshreddy Gurala, Abhishek D Polavarapu, Pretty Sara Idiculla, Vivek Gumaste

**Affiliations:** 1 Gastroenterology and Hepatology, Staten Island University Hospital, Northwell Health, Staten Island, USA; 2 Medicine, Sree Gokulam Medical College and Research Foundation, Trivandrum, IND; 3 Gastroenterology, Staten Island University Hospital, Northwell Health, Staten Island, USA

**Keywords:** inadvertent dislodgement, gi endoscopy, savary dilator, percutaneous endoscopic gastrostomy removal, percutaneous endoscopic gastrostomy (peg) feeding

## Abstract

Inadvertent removal or dislodgement is the most commonly encountered complication in patients with percutaneous endoscopic gastrostomy (PEG) tube. Once the gastrocutaneous fistula is formed, bedside tube replacement can be performed at the same site, within 24 hours of dislodgement. Usually, after this timeframe, the tract closes; hence, it is recommended to perform a replacement at a different site. We report a case of a 52-year-old female who presented after 24 hours of inadvertent PEG tube removal. A replacement was performed successfully via endoscopy at the same site of the erstwhile PEG tube, although it appeared to be closed.

## Introduction

Gastrostomy is the preferred route of feeding in patients who require long-term enteral nutrition. With the advent of endoscopy, percutaneous endoscopic gastrostomy (PEG) has overcome surgical gastrostomy due to its cost-effectiveness and less invasive nature along with fewer complications. Inadvertent removal or dislodgement is the most encountered complication in patients with PEG tubes. The reported incidence in some studies is as high as 12.8% [1]. In a patient with a PEG tube dislodgement, less than four weeks old, endoscopic-guided replacement at a different site with surgical evaluation is the preferred approach [2,3]. After four weeks, bedside replacement can be performed at the same site, within 24 hours of dislodgement as the gastrocutaneous fistula is well matured. Usually, after this timeframe, the tract closes; hence, it is recommended to perform a replacement at a different site. Herein, we present a case of a 52-year-old female who presented to us 24 hours after inadvertent removal of PEG tube, and a replacement was performed successfully via endoscopy at the same site, which appeared to be closed.

This case was presented as a poster at the ACG Gastroenterology Conference in October 2020 (https://journals.lww.com/ajg/Fulltext/2020/10001/S2076_A_Unique_Case_of_PEG_Tube_Replacement.2076.aspx).

## Case presentation

A 52-year-old female presented to our emergency department 24 hours after accidental dislodgement of the PEG tube. Her previous medical history includes PEG tube placement a month ago for oropharyngeal dysphagia secondary to anoxic brain injury. Her vital signs were stable on arrival. On physical examination, the abdomen was soft and non-tender without any guarding or rigidity. The PEG tube site appeared clean and closed. Basic laboratory evaluations, including a complete blood count and international normalized ratio (INR), were found to be within normal limits.

A decision was made to replace the PEG tube via endoscopy at a different site after COVID-19 testing according to our institutional protocol. On day three, the COVID-19 PCR came back negative, and esophagogastroduodenoscopy was performed under conscious sedation. The esophagus and duodenum appeared normal, and the previous PEG tube site in the stomach was noted.

**Figure 1:**
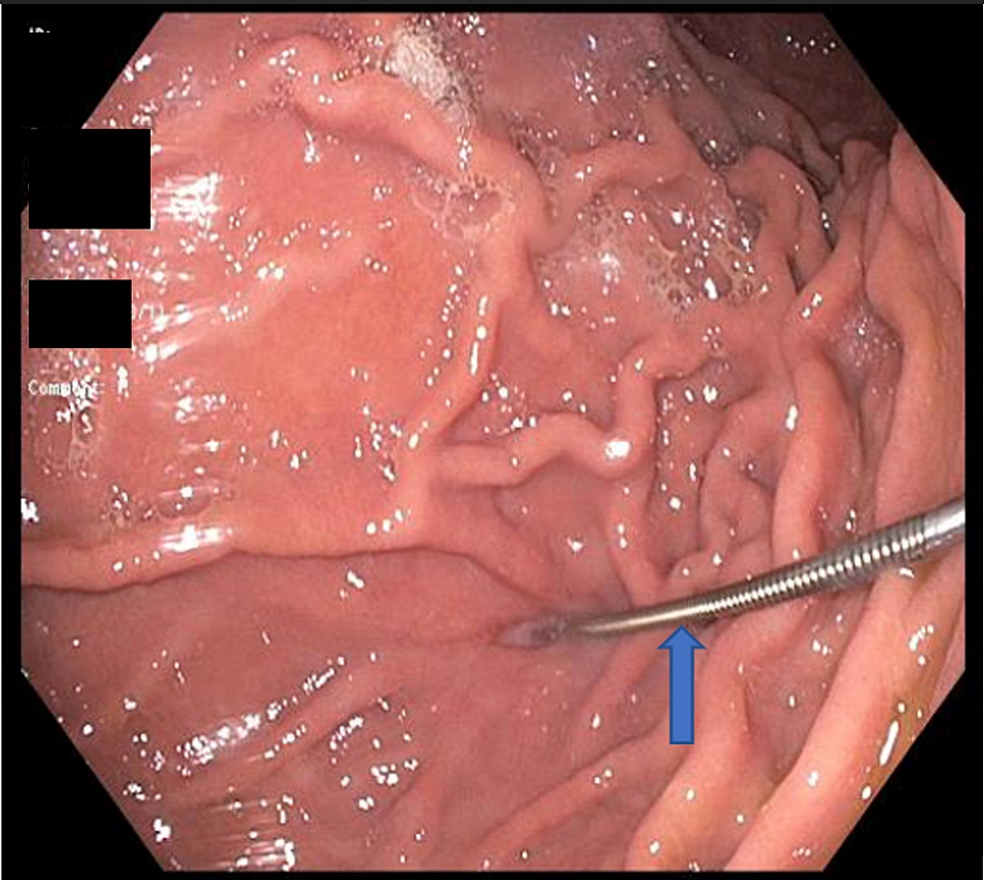
Jagwire in the gastric lumen at the previous PEG site (arrow).

**Figure 2:**
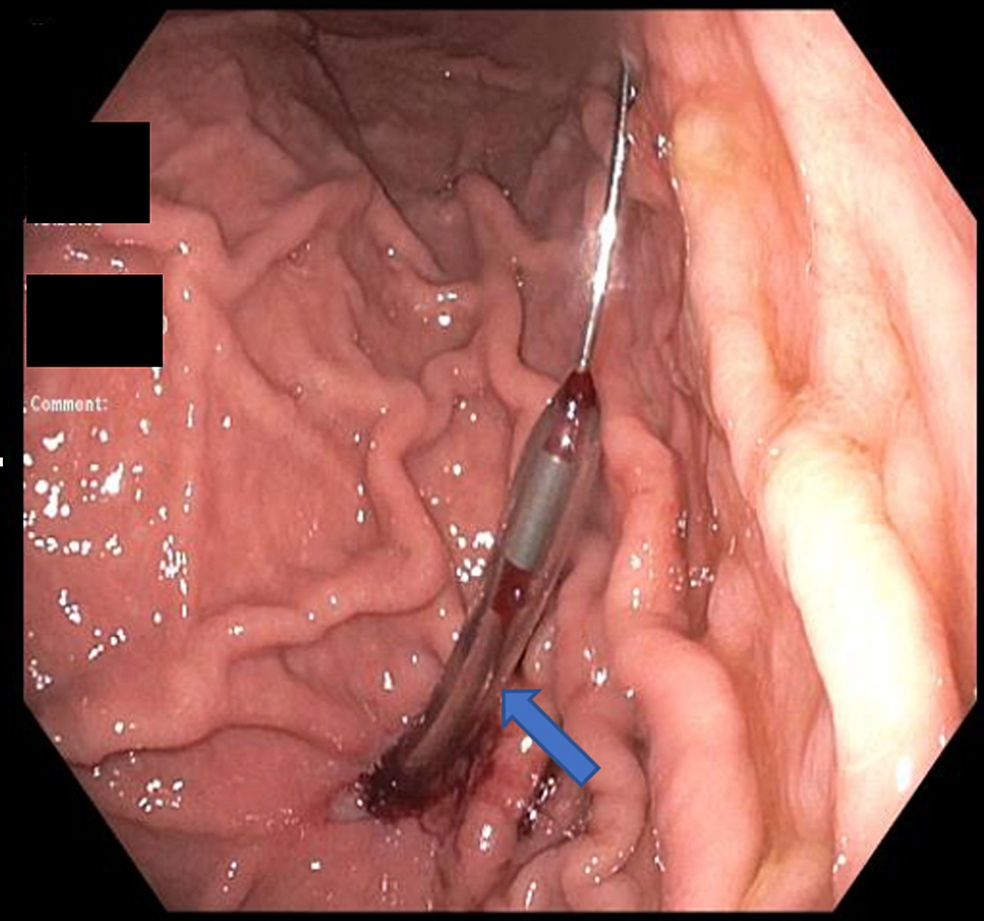
Savary dilator introduced over the jagwire under direct endoscopic vision (arrow).

Although the external site appeared well healed, we could introduce a jagwire through a minute opening with minimal force into the gastric lumen under direct endoscopic vision. A 20F balloon PEG tube was introduced over the guidewire but failed to reach the lumen due to buckling. A 26F Savary dilator was used to dilate the tract but was still unsuccessful. We attempted a novel technique where a 26F Savary dilator was advanced over the jagwire, followed by the removal of the wire and keeping the dilator in place (Figures 1, 2).

**Figure 3:**
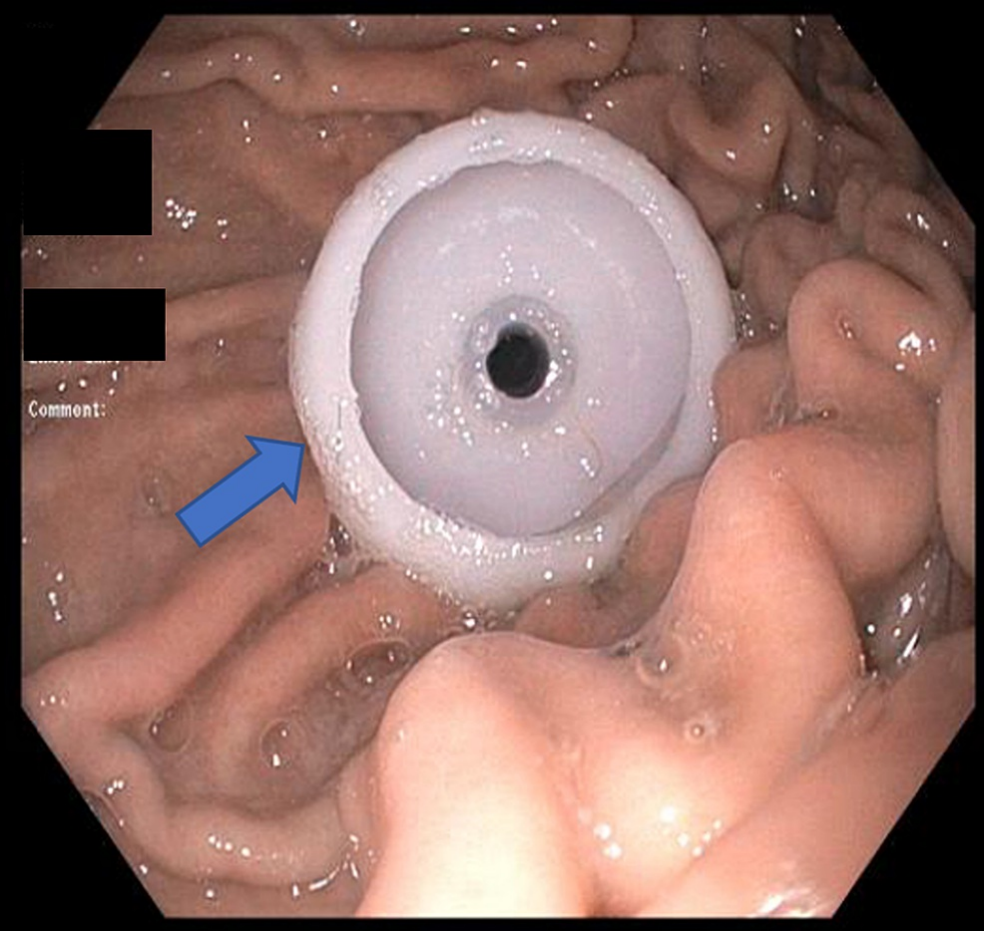
Internal bolster location confirmed endoscopically after successful PEG tube placement (arrow).

A PEG insertion wire was then introduced through the dilator and advanced until visible in the gastric lumen. Then, the PEG insertion wire was caught by the retrieval snare passed through the endoscope and brought out through the mouth. A bolstered PEG tube was secured to the peg insertion wire and pulled through the abdominal wall in the same way as the “pull technique.” A satisfactory final position was confirmed endoscopically (Figure 3).

The gastrostomy tube was secured with the outer flange positioned at 4 cm. On two months follow-up, the PEG tube was functioning adequately without any complications.

## Discussion

PEG was first introduced in 1980 as an alternative to the surgical placement of feeding tubes in patients requiring long-term nutrition [4]. Since then, it grew rapidly and became the second most common indication for upper endoscopy with more than 215,000 PEG placements annually [5]. Although generally considered a safe procedure with low morbidity and a success rate of 95%, the potential complications cannot be overlooked. Among them, one of the most common is inadvertent removal or dislodgement [6]. The reason being many gastrostomy tubes can be easily removed with external traction of 10-14 pounds. The replacement strategy depends upon when the initial PEG was placed and the timing of presentation after the accidental dislodgement. Most gastrostomy tract matures by two weeks after placement, although this process can take up to one month or even longer in immunosuppressed and malnourished patients [7]. Hence, if the tube is less than four weeks old, the tract should be allowed to heal for 7-10 days before a new PEG tube is considered at a different site [7,8]. In dislodgement of the tube one month after placement, we can assume that the gastrocutaneous tract is formed, and replacement can be done without endoscopy at the bedside. However, once the tube is out, the tract starts to close as early as eight hours and mostly by 24 hours [9]. Hence, after 24 hours, experts recommend performing endoscopic insertion rather than a blind forceful attempt to place the PEG tube to prevent intraperitoneal placement.

As in our case, the patient presented after 24 hours, and the gastrostomy tract appeared to be closed. Even then, we successfully inserted the jagwire with minimal resistance through a minute opening on the previous site.

The jagwire, one of the guidewires used for biliary cannulation, has these unique characteristics of stiffness, hydrophilic coating, and pushability [10]. These features allow easy navigation through tortuous anatomy and likely assisted the passage of wire through the gastrostomy tract.

The Savary dilator has a tapered blunt end unlike the trocar needle, and its lumen allows a direct tract to pass the PEG insertion wire into the stomach, which was not otherwise possible.

## Conclusions

In conclusion, inadvertent removal of the PEG tube is the most frequent complication of PEG, and all gastroenterologists should be well aware of the management. Our case emphasizes that once the gastrostomy tract is mature, an endoscopic replacement can be performed safely at the same site even after 24 hours using a jagwire and a Savary dilator. A simple utilization of these endoscopic accessories can avoid a new skin wound, gastric puncture, and its potential complications.
